# Miniopen Oblique Lateral L5-S1 Interbody Fusion: A Report of 2 Cases

**DOI:** 10.1155/2014/603531

**Published:** 2014-10-21

**Authors:** Keijiro Kanno, Seiji Ohtori, Sumihisa Orita, Kazuyo Yamauchi, Yawara Eguchi, Yasuchika Aoki, Junichi Nakamura, Masayuki Miyagi, Miyako Suzuki, Gou Kubota, Kazuhide Inage, Takeshi Sainoh, Jun Sato, Yasuhiro Shiga, Koki Abe, Kazuki Fujimoto, Hiroto Kanamoto, Tomoaki Toyone, Gen Inoue, Eiji Hanaoka, Kazuhisa Takahashi

**Affiliations:** Department of Orthopaedic Surgery, Graduate School of Medicine, Chiba University, 1-8-1 Inohana, Chuo-ku, Chiba 260-8670, Japan

## Abstract

Extreme lateral interbody fusion (XLIF) has been widely used for minimally invasive anterior lumbar interbody fusion (ALIF), but an approach to L5-S1 is difficult because of the iliac crest. In the current study, we present 2 cases using minimally invasive oblique lateral interbody fusion (OLIF) of L5-S1. The patients showed foraminal stenosis between L5 and S1 and severe low back and leg pain. The patients were placed in a lateral decubitus position and underwent OLIF surgery (using a cage and bone graft from the iliac crest) without posterior decompression. Posterior screws were used in the patients. Pain scores significantly improved after surgery. There was no spinal nerve, major vessel, peritoneal, or urinary injury. OLIF surgery was minimally invasive and produced good surgical results without complications.

## 1. Introduction

Open approaches such as anterior lumbar interbody fusion (ALIF), posterior lumbar interbody fusion (PLIF), and transforaminal lumbar interbody fusion (TLIF) have been reported to have high rates of success for lumbar spinal fusion [1–3], although intraoperative concerns and iatrogenic complications are known [4, 5].

The minimally invasive lateral transpsoas approach to the lumbar spine is also known as extreme lateral interbody fusion (XLIF) [6, 7]. The advantages of XLIF include minimally invasive access to the lumbar spine, less blood loss compared with open surgery, decreased operative times, shorter hospital stays, and less postoperative pain [6]. XLIF is unique in that it can be used to gain access to the lumbar spine via a lateral approach by splitting the center psoas major muscle. In this regard, the approach to L5-S1 is extremely difficult because of the presence of the iliac crest.

Miniopen oblique lumbar interbody fusion (OLIF) was reported in 2012 [8]. In this procedure, a 4 cm skin incision is made 6–10 cm anterior from the midportion of an intervertebral disk; the approach is made between the peritoneum and the psoas muscle. A banana-shaped polyetheretherketone cage (Boomerang; Medtronic, Inc., Minneapolis, MN, USA) filled with a bone graft was used in the reported study [8]. However, the authors mentioned that the approach to L2–L5 is easy, but another approach might be preferred at L5-S1 because of the risks associated with mobilization of the iliac vessels and because the presence of the iliac wing disturbed insertion of the cage [8]. Subsequently, Medtronic developed their OLIF system (OLIF25) using a new device, and this procedure enables the placement of a larger interbody graft into the disk space for anterior column support and segmental sagittal alignment, while minimizing the nerve, muscle, and bone obstacles associated with traditional direct lateral approaches. This system can be applied to L5-S1 between major vessels using a lateral decubitus operating position. However, to our knowledge, results of this OLIF surgery have not been reported.

The purpose of the current study was to present 2 cases using OLIF of L5-S1 in a lateral decubitus position.

## 2. Case Presentation

The protocols for human procedures used in this study were approved by the ethics committee of our institution. Written, signed informed consent was received from each patient before treatment.

### 2.1. Case  1

**Figure 1:**
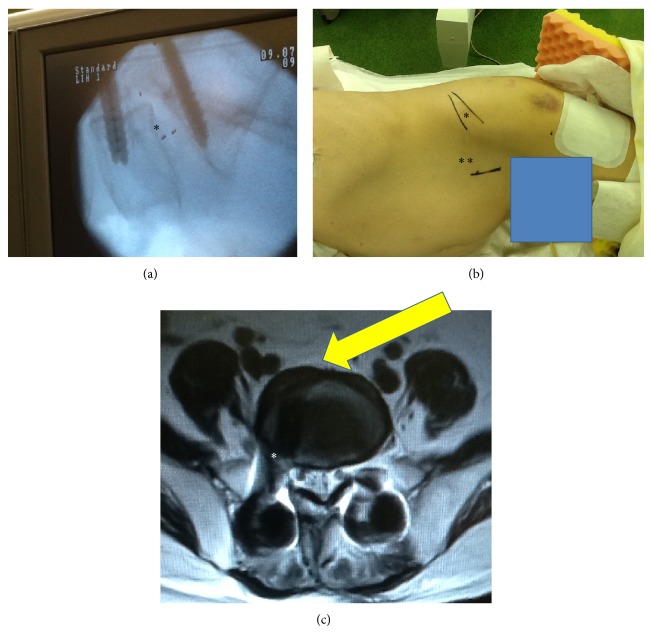
(a) Posterior movement of the TLIF cage 6 months after surgery (^*^). (b) The patient was placed in a lateral decubitus position. ^*^Midportion of the L5-S1 disk. ^**^A 5 cm skin incision was made 10 cm anterior from the midportion of the L5-S1 disk. (c) MRI showing right foraminal stenosis between L5 and S1 because of the cage. Arrow indicates the approach between major vessels.

**Figure 2:**
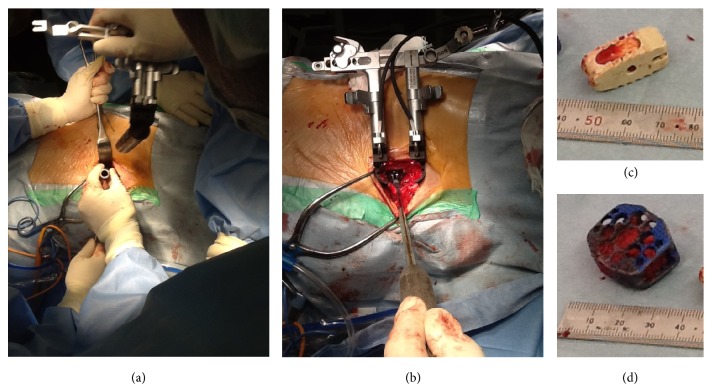
((a) and (b)) Dilators were used at first, and, next, retractors with light were used. (c) Removed cage. (d) Insertion of the SynCage.

**Figure 3:**
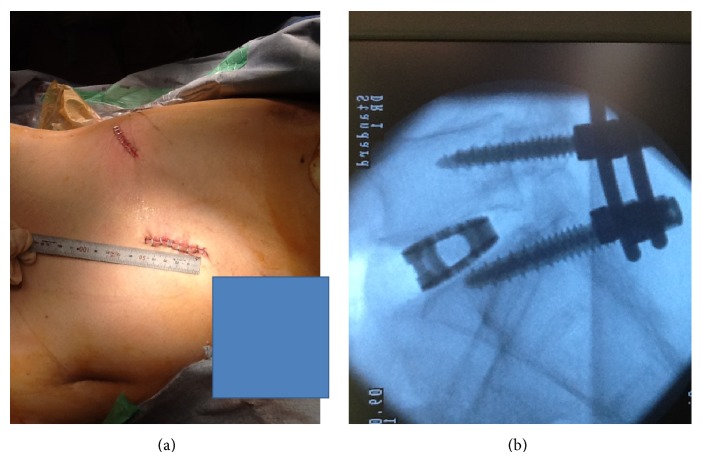
(a) Sutured skin after approaching L5-S1 and harvesting the iliac bone. (b) X-ray image after surgery.

A 68-year-old man presented with a 15-month history of right sciatica with complaints of pain in his buttocks and the lateral aspect of his right leg corresponding to the L5 dermatome. We performed TLIF with instrumentation at L5-S1. The patient became symptom-free immediately after surgery. However, 6 months later, the cage had moved posteriorly (Figure 1(a)). The patient showed severe leg pain corresponding to the right L5 spinal nerve. MRI revealed right foraminal stenosis between L5 and S1 because of the cage. We performed OLIF with the patient in a lateral decubitus position. A 5 cm skin incision was made 10 cm anterior from the midportion of the L5-S1 disk on the left side, and the retroperitoneal space was accessed (Figure 1(b)). The L5-S1 disk was accessed using an OLIF device between the major vessels (Figures 1(c), 2(a), and 2(b)). First, dilators were used, and then retractors with light were used (Figures 2(a) and 2(b)). The TLIF cage was removed and we inserted a SynCage (Synthes, Inc., PA, USA) filled with bone graft harvested from the iliac crest (Figures 2(c) and 2(d)). We used additional percutaneous pedicle screws. X-ray image examination showed good stability and disk height (Figure 3). On a follow-up examination, the patient was symptom-free.

### 2.2. Case  2

**Figure 4:**
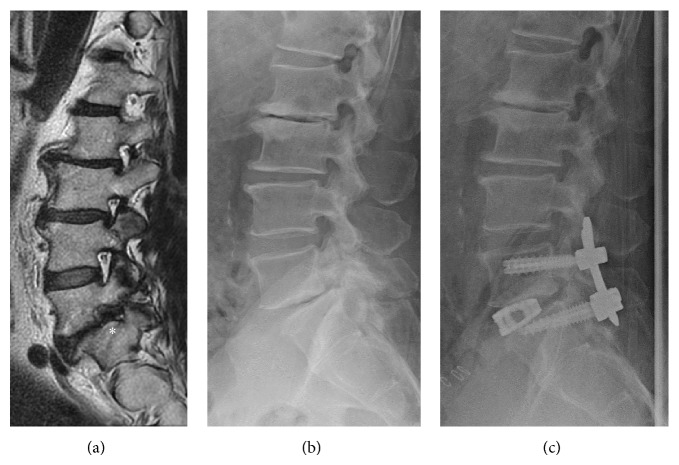
(a) Sagittal T2-weighted MRI showing foraminal stenosis at L5-S1 (^*^). (b) X-ray image showing L5-S1 disk narrowing and L5 spondylolytic spondylolisthesis before surgery. (c) X-ray image after surgery.

A 58-year-old man presented with a 12-month history of bilateral sciatica with complaints of pain in his buttocks and the lateral aspects of his legs. There was no apparent motor weakness. Sensory examination confirmed hypoalgesia in the lateral aspect of his right lower leg corresponding to the L5 dermatome. An X-ray image examination showed L5-S1 disk narrowing and L5 spondylolytic spondylolisthesis, and MRI revealed severe foraminal stenosis at L5-S1 on T2-weighted imaging (Figures 4(a) and 4(b)). Because conservative treatment was not effective, surgery was planned. We performed an OLIF with the patient in a lateral decubitus position. Using the same procedure described for case 1, a 5 cm skin incision was made 10 cm anterior from the midportion of the L5-S1 disk on the left side, and the retroperitoneal space was accessed. The L5-S1 disk was accessed using an OLIF device between the major vessels. The intervertebral disk was removed, and a SynCage (Synthes, Inc.) filled with bone graft harvested from the iliac crest was inserted. We used additional percutaneous pedicle screws. X-ray image examination showed good stability and disk height (Figure 4(c)). On a follow-up examination, the patient was symptom-free.

## 3. Discussion

In the current study, we evaluated an approach using OLIF for L5-S1 with the patient in a lateral decubitus position. There were no complications during surgery. Low back and leg pain significantly decreased after surgery.

Recently, a minimally invasive lateral transpsoas approach to the lumbar spine, known as XLIF, has been used. This method has been used for degenerative lumbar disease, discogenic back pain, and kyphoscoliosis. Changes in coronal and sagittal plane alignment following XLIF for degenerative scoliosis have been reported [9–11]. Excellent results, less blood loss, shorter length of hospital stay, and a lower incidence of infection compared with traditional methods were reported [9–11]. Several authors have reported indirect decompression using a stand-alone XLIF cage without posterior decompression or pedicle screws. A total of 84 stand-alone XLIF or 21 stand-alone XLIF patients were evaluated and they showed evidence of solid arthrodesis and improvements in pain scores [12, 13]. However, XLIF is unique in the way that access to the lumbar spine is obtained via a lateral approach by splitting the center psoas major muscle. In this regard, it is extremely difficult to approach L5-S1 because of the presence of the iliac crest.

Silvestre et al. reported a retrospective study including 179 patients who underwent miniopen OLIF at one institution [8]. The patient was placed in a lateral decubitus position because they approached L1-S1 between the major vessels and the psoas muscle. The procedure was performed on the lumbar spine at L1-L2 in 4, L2-L3 in 54, L3-L4 in 120, L4-L5 in 134, and L5-S1 in 6 patients. The number of patients with OLIF at L5-S1 was small because of the difficulty of retracting the iliac vein and ascending lumbar vein. Silvestre et al. concluded that another approach might be preferred at L5-S1 because of the risks associated with mobilization of the vessels and the presence of the iliac wing. Recent cadaveric study revealed that the OLIF method could approach to the L2-S1 discs in patients at a lateral decubitus position. Minimal psoas retraction without significant tendon disruption allowed for a generous corridor to the disc space. The L5-S1 disc space can be accessed from an oblique angle consistently with gentle retraction of the iliac vessels [14].

In the current study, although the patient was placed in a lateral decubitus position, we could access L5-S1 using a new retractor and device. We approached L5-S1 between major vessels, and there was a lower risk of injury to the iliac vein and ascending lumbar vein. We concluded that this procedure is a safe approach to L5-S1. Another merit of this approach was that if the surgeon needs to access disks at multiple levels, he can perform interbody fusion from L1 to S1 with the patient in a lateral decubitus position.

In the current study, we used posterior fixation. Stand-alone short-segment (1- or 2-level) XLIF was investigated in 98 patients. While significant gains in segmental lumbar lordosis and disk height were observed overall, some patients experienced less improvement because of a higher rate of interbody graft subsidence [15]. Subsidence was correlated with transient clinical worsening [15]. In the current study, we used posterior fixation to improve fusion rate and avoid revision surgery or cage subsidence.

In conclusion, OLIF of L5-S1 was minimally invasive and produced good surgical results without complications.
